# Total flow intensity, active flow intensity and volume related flow intensity as new quantitative metrics in optical coherence tomography angiography

**DOI:** 10.1038/s41598-021-88681-y

**Published:** 2021-04-27

**Authors:** Alessandro Arrigo, Cristian Perra, Emanuela Aragona, Daniele Giusto, Francesco Bandello, Maurizio Battaglia Parodi

**Affiliations:** 1 Department of Ophthalmology, IRCCS Ospedale San Raffaele, University Vita-Salute, via Olgettina 60, 20132 Milan, Italy; 2 CNIT Research Unit, Department of Electrical and Electronic Engineering (DIEE), University of Cagliari, Cagliari, Italy

**Keywords:** Medical research, Biomarkers, Translational research

## Abstract

Optical coherence tomography (OCT) angiography (OCTA) is a non-invasive tool for the in-vivo study of the intraretinal vascular network. It is based on the analysis of motion particles within the retina to reconstruct the paths followed by the erythrocytes, i.e. retinal capillaries. To date, qualitative and quantitative information are based on the morphological features disclosed by retinal capillaries. In the present study, we proposed new quantitative functional metrics, named Total Flow Intensity (TFI), Active Flow Intensity (AFI), and Volume-related Flow Intensity (VFI), based on the processing of the blood flow signal detected by OCTA. We studied these metrics in a cohort of healthy subjects, and we assessed their clinical utility by including a cohort of age-matched patients affected by Stargardt disease. Moreover, we compared TFI, AFI, and VFI to the widely used vessel density (VD) parameter. TFI, AFI, and VFI were able to describe in detail the different properties of the retinal vascular compartment. In particular, TFI was intended as the overall amount of volumetric retinal blood flow. AFI represented a selective measure of voxels disclosing blood flow signal. VFI was developed to put in relationship the volumetric blood flow information with the not vascularized retinal volume. In conclusion, TFI, AFI, and VFI were proposed as feasible functional OCTA biomarkers based on the analysis of retinal blood flow signal.

## Introduction

Optical coherence tomography (OCT) angiography (OCTA) represented a turning point for the non-invasive analysis of intraretinal vascular network, in vivo, in human eyes. OCTA output is obtained by the acquisition of 70,000 A-scans/sec for spectral domain OCT technology or 100,000 A-scans/sec for swept source OCT one, through acquisition volumes covering the entire macular region, from 3 × 3 mm scans up to 12 × 12 mm scans^1,2^. The theorical principle is based on the calculation of a decorrelation signal between timely consecutive A-scans detecting intraretinal motion particles, interpreted as the erythrocytes moving toward intraretinal capillaries. The detected final signal mainly depends both from the speed and the amount of motion particles within each voxel^1–3^.

The final output is the tridimensional reconstruction of the paths followed by these motion particles, namely intraretinal capillaries. The current OCTA resolution is approximatively of 8 µm, guaranteeing a very good detection of a big amount of intraretinal capillaries. However, it should be always noted that the larger is the acquisition volume, the lower will be the resolution. For this reason, 3 × 3 mm scans currently offer the greatest resolution, although limited on a small field of view^1,2^.

OCTA is currently used to obtain the morphological representation of intraretinal vascular network, with particular regards to deeper capillaries, which cannot be detected by conventional dye-based angiographies, because of leakage masking effect. The starting approach to OCTA datasets was the qualitative evaluation of the morphological status of intraretinal capillaries. In a second step, several authors have proposed different metrics based on the quantification of given morphological properties of the OCTA reconstructions, in the attempt to interpret in a more objectively way the characteristics of intraretinal vessels^4–13^.

Vessel density made somehow a revolution in the analysis of OCTA data; however, it discloses two main limitations. The first one is the complete dependence from the proper morphological representation of intraretinal capillaries. The second one is related to the high sensitivity in detecting a loss of capillaries, but the extremely poor specificity in providing specific quantitative features for each retinal disease. Indeed, vessel density results decreased almost in all retinal diseases.

More recently, other parameters have been introduced to improve the sensitivity of OCTA in detecting vascular alterations, namely vessel tortuosity, vessel dispersion and vessel rarefaction. These latter represented a way to provide indirect functional information starting from a morphological output^4–13^.

However, also these parameters are bound by the quality of final vascular plexa reconstructions and their morphology. Overall considering all these features, OCTA analysis can be nowadays considered a three-steps procedure. The first step is the acquisition of functional information, namely the motion signal. Then, the second step is to convert this functional information into a morphological one, namely the reconstructed vessels. Finally, the third step is to adopt morphological metrics to indirectly obtain functional vascular information.

One of the most intriguing perspectives of OCTA analyses is the possibility to remarkably improve the amount of information acquired by the development of new post-processing algorithms.

This opportunity may make it possible to overcome the current three-steps to reach the final OCTA information. This is the reason why, in the present study, we proposed new OCTA quantitative parameters, based on the direct analysis of the OCTA output obtained after the calculation of the decorrelation signal related to intraretinal blood flow.

## Materials and methods

The study was design as feasibility, case-series investigation. We recruited healthy volunteers and patients with genetically confirmed (ABCA4 mutation) diagnosis of Stargardt disease (STGD) from the Ophthalmology Unit of San Raffaele Hospital, Milan, Italy. Each subject and patient provided signed informed consent before the examination. The study was conducted in accordance with the Declaration of Helsinki and it was approved by the Ethical Committee of the Vita-Salute San Raffaele University of Milan. Inclusion criteria were age > 18 years old, unremarkable ophthalmologic history for healthy subjects, confirmed diagnosis of STGD for the patients. Exclusion criteria were refractive errors greater than ± 1.5D, increased media opacities, any type of retinal and/or optic nerve diseases, any ophthalmic surgery in the last six months, any systemic conditions potentially affecting the results of the study. Moreover, we excluded STGD patients with severe stages of the disease (BCVA < 20/200), to reduce possible biases related with extremely low fixation. We included STGD patients in order to make possible a reliable age-matched comparison with our healthy volunteers, thus considering datasets made by young age samples. All the subjects and the patients underwent a complete ophthalmologic examination, including best corrected visual acuity (BCVA) using standard Early Treatment Diabetic Retinopathy Study charts, slit-lamp evaluation of anterior and posterior segments and intraocular pressure Goldmann applanation tonometry. SS-OCT DRI Topcon Triton (Topcon Corporation; Tokyo, Japan) was used to perform OCTA acquisitions; we obtained 3 × 3 mm high-resolution images centered on the macular region. Eye-tracking was used to assess possible fixation loss. Only high-quality images, evaluated by means of Topcon index quality (> 80), were considered. OCTA images were analyzed by the Topcon full spectrum amplitude decorrelation angiography algorithm. Each patient contributed with a single eye, which was randomly selected.

The common nomenclature followed for identifying the intraretinal vascular plexa composing the entire intraretinal vascular network is based on the segmentation of different layers. In particular, the superficial vascular complex (SVC) is comprised between the inner limiting membrane (ILM) and the inner plexiform layer-inner nuclear layer (IPL-INL) interface, whereas the deep vascular complex (DVC) is included between the IPL-INL and the outer plexiform layer-outer nuclear layer (OPL-ONL) interfaces^14^.

**Figure 1:**
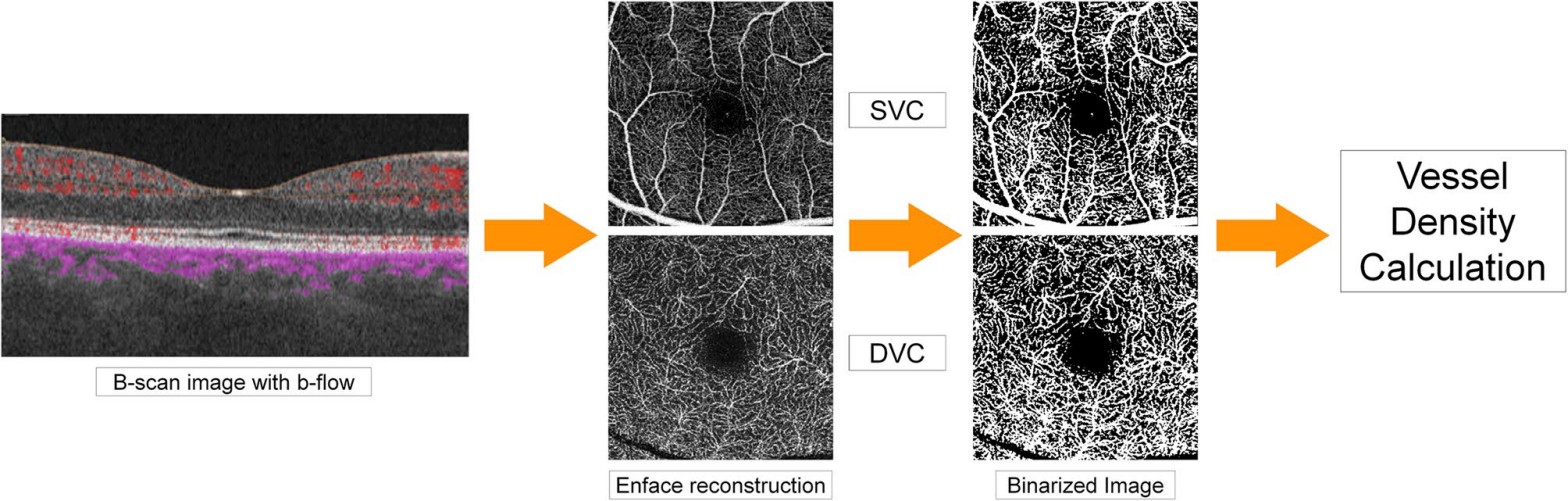
Graphical steps for vessel density calculation. *SVC* superficial vascular complex, *DVC* deep vascular complex.

**Figure 2:**
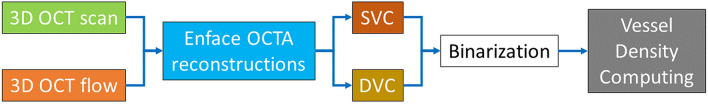
Workflow for computing vessel density OCTA metric. *OCT* optical coherence tomography, *OCTA* optical coherence tomography angiography, *SVC* superficial vascular complex, *DVC* deep vascular complex.

For vessel density (VD) calculation, we considered superficial (SVC) and deep (DVC) capillary vascular complexes. In particular, we exported SVC and DVC automatic segmentations, which were carefully checked by an expert ophthalmologist (AA) and eventually manually corrected. These images were loaded in ImageJ^15^. Each segmentation was binarized through a mean threshold. Foveal avascular zone was manually segmented and considered as exclusion criterion. Then, we applied in-house scripts to calculate the ratio between white and black voxels, namely VD (Figs. 1 and 2).

Volumetric blood flow information acquired from OCTA were processed in order to compute our proposed novel functional metrics characterizing the retinal blood flow. The theoretical assumption was that the higher was the reflectivity of intraretinal voxels resulting positive on OCTA for motion particles, the higher was the blood flow signal. This signal may depend on the amount of particles passing within the voxels and/or the speed of these particles, resulting within the range of sensitivity of the OCTA device.

We considered SVC and DVC for computing three novel metrics named “Total Flow Intensity” (TFI), “Active Flow Intensity” (AFI), and “Volume-related Flow Intensity” (VFI). As shown in Fig. 2, the procedure is composed of two main steps: OCTA segmentation and metrics computation. Adjacent OCT scans are often misaligned resulting in a missing correspondence between adjacent voxels in the z direction. The procedure of spatial alignment of OCT scans is called registration.

There exist several algorithms in literature for medical image registration. Considering that it is important to assure that the registration process does not alter the voxel’s intensity, a basic algorithm based only on rigid translation is adopted. The layer segmentation algorithm is based on mathematical morphology and voxel clustering techniques presented in and summarized as follows.

**Figure 3:**
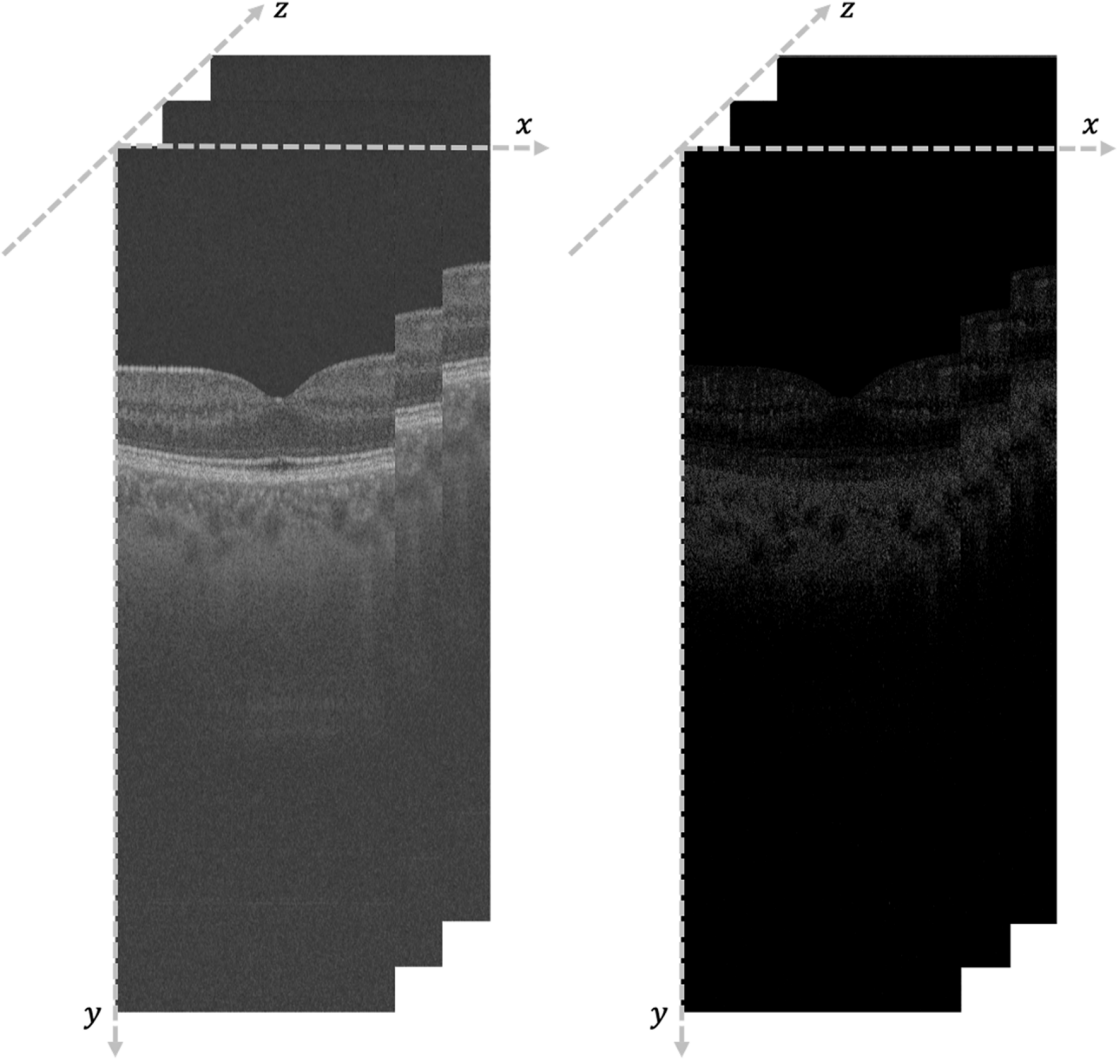
Illustrative case of the coordinate reference system and a visual example of corresponding slices from OCT scan and OCT flow.

Let us denote by S(x,y,z) the volume of the x–y OCT scans acquired along the z-direction and denote by F(x,y,z) the corresponding OCT flows. Figure 3 shows an example of the coordinate reference system and a visual example of corresponding slices from OCT scan and OCT flow. A global threshold $t$ is computed applying the Otsu’s method to the volume $S$.

The scan volume is then thresholded as follows

$${S}^{^{\prime}}\left(x,y,z\right)=\left\{\begin{array}{cc}S\left(x,y,z\right)& S\left(x,y,z\right)\ge t\\ 0& S\left(x,y,z\right)<t\end{array}\right.$$

The barycenter $\mathbf{b}=({b}_{x},{b}_{y},{b}_{z})$ computed from ${S}^{^{\prime}}\hspace{0.25em}(x,y,z)$ is then used for a rigid translation of the per-slice barycenter ${\mathbf{b}}_{\mathbf{x}\mathbf{y}}(z)$ to the barycenter $\mathbf{b}$. Finally, the best motion vector between adjacent slices is computed constrained by the minimization of the mean square error. The motion vector search area is controlled by the two parameters $\Delta x$, and $\Delta y$, for the full search area $[-\Delta x,\Delta x]\times [-\Delta y,\Delta y]$. The same translations are applied to corresponding frames in the OCTA flow volume $F(x,y,z)$. Finally, zero-padding is applied to the new space around the edges of the slices generated by the rigid translation process.

The next step consists in computing the retinal layers. The layer detection process is composed of different steps. Each slice of the OCT volume is thresholded using the Otsu’s method. Then a sequence of morphological transformation is applied for obtaining a clean mask of the region of interest. These steps are erosion, closing, filling, removing small unconnected regions, and cleaning the left and right sides of the image.

**Figure 4:**
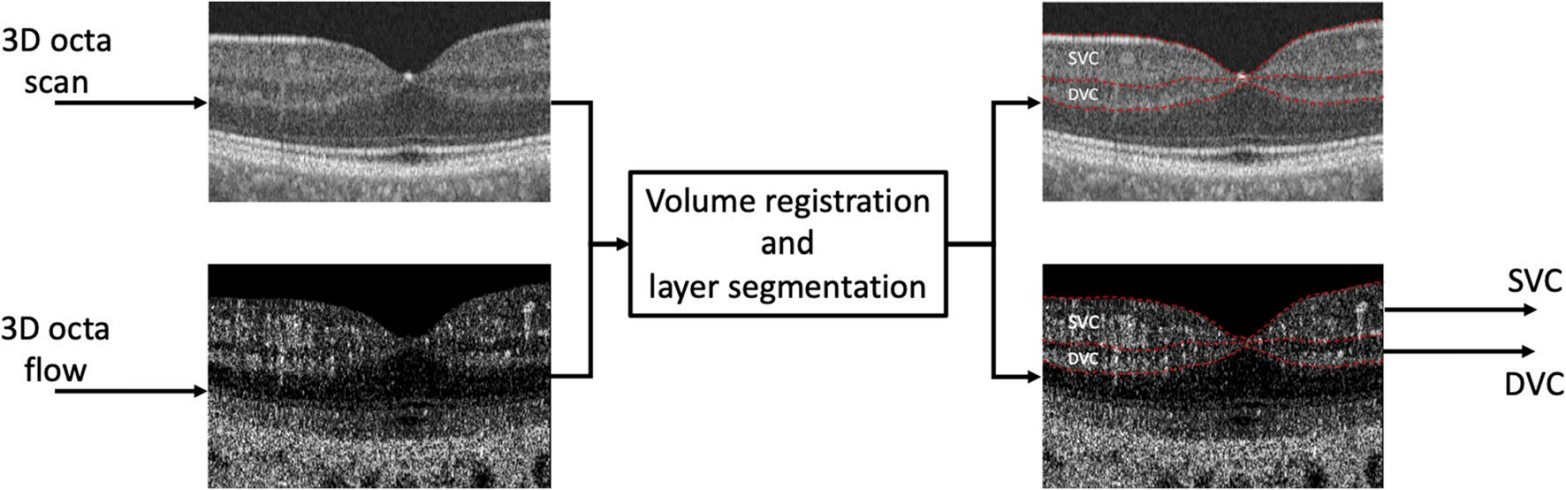
Schematic representation of volume registration and layer segmentation.

The layers of the OCT are computed using a multi-layer graph search based as proposed in and applied to OCT signals in that computes a set of surfaces across voxels’ boundaries. From the obtained surfaces only the ones corresponding to the ILM, the IPL-INL interface, and the IPL-INL interface have been since they provide the boundaries for the SVC and DVC volumes (Fig. 4). The obtained layers have been then visually inspected, and the incorrect ones manually adjusted.

**Figure 5:**
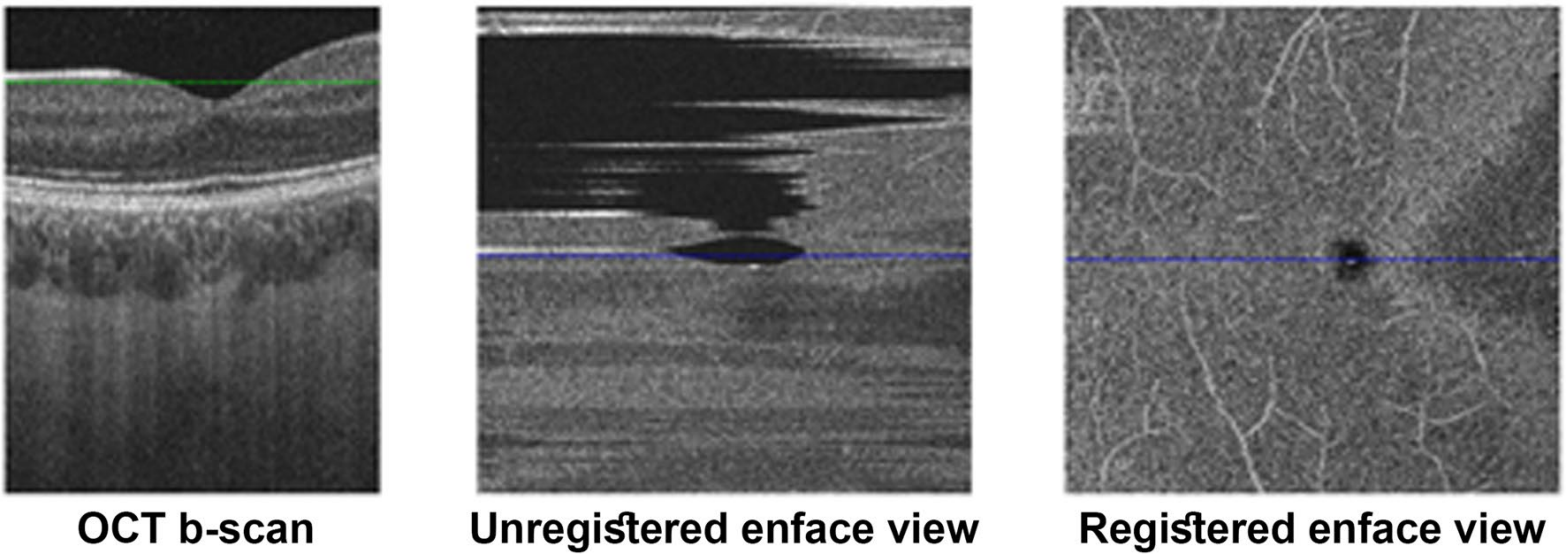
Example of volume registration. The left image shows the front view of a b-scan OCT foveal slice (z = 166) corresponding to the blue line in enface views. The central image represents the original enface view of a plane (y = 84) corresponding to the plane cut by the green line in b-scan OCT. Right image shows the same enface view after volume registration.

Each OCTA volume is exported as raw data from the OCTA acquisition device as a set of 320 grayscale images (8-bit per pixel) with resolution 320 × 992 pixels. These images are not aligned and a registration process is required for matching corresponding regions in adjacent images (Fig. 5). The entire process generates a segmentation of the OCTA volumetric information (3D OCT scan and 3D OCT flow) from which the SVC and the DVC retinal layers are extracted. These two layers are fed as input to the metrics computation blocks that produce the proposed TFI, AFI, and VFI metrics for SVC (${S}_{TFI}$, ${S}_{AFI}$, ${S}_{VFI}$) and for DVC (${D}_{TFI}$, ${D}_{AFI}$, ${D}_{VFI}$). The algorithm built for processing the OCTA volume and computing the proposed metrics has been implemented in Matlab Software Package (The Mathworks Inc., Matlab, R2020a).

The TFI metric is a measure of the amount of volumetric blood flow in SVC or DVC. It is computed as the sum of the squared voxel flow intensity ($f(x,y,z)$) using the formula

$${I}_{TFI}=\frac{1}{{w}_{TFI}V}\sum_{i=1}^{V}f{\left({x}_{i},{y}_{i},{z}_{i}\right)}^{2}$$

where $V$ is the number of voxels $P\left({x}_{i},{y}_{i},{z}_{i}\right)$ in the retinal layer under test, $f({x}_{i},{y}_{i},{z}_{i})$ is the blood flow intensity of these voxels, ${w}_{TFI}$ is scaling parameter introduced for controlling the index range. The theoretical range for the TFI metric is ${I}_{TFI}\epsilon ]\mathrm{0,1}[$. When ${I}_{TFI}\to 0$, the retinal layer does not exhibit flow activity, while when ${I}_{TFI}\to 1$, there is very intense flow activity.

The AFI metric is a measure of the average flow over all ‘active’ voxels. It is computed using the formula

$${I}_{AFI}=\frac{1}{{w}_{\mathrm{AFI}}F}\sum_{i=1}^{F}f{\left({x}_{i},{y}_{i},{z}_{i}\right)}^{2}$$

where $F$ is the number of voxels $P\left({x}_{i},{y}_{i},{z}_{i}\right)$ in the retinal layer under test where $f\left(P\right)>0$ (i.e. the OCT blood flow exhibits some activity), $f({x}_{i},{y}_{i},{z}_{i})$ is the blood flow intensity of these voxels, ${w}_{C}$ is scaling parameter introduced for controlling the index range. The theoretical range for the AFI is ${I}_{TFI}\epsilon ]\mathrm{0,1}[$. When ${I}_{TFI}\to 0$, the blood vessels in the layer under test do not exhibit flow activity, while when ${I}_{TFI}\to 1$, the blood vessels exhibit very intense flow activity.

**Figure 6:**
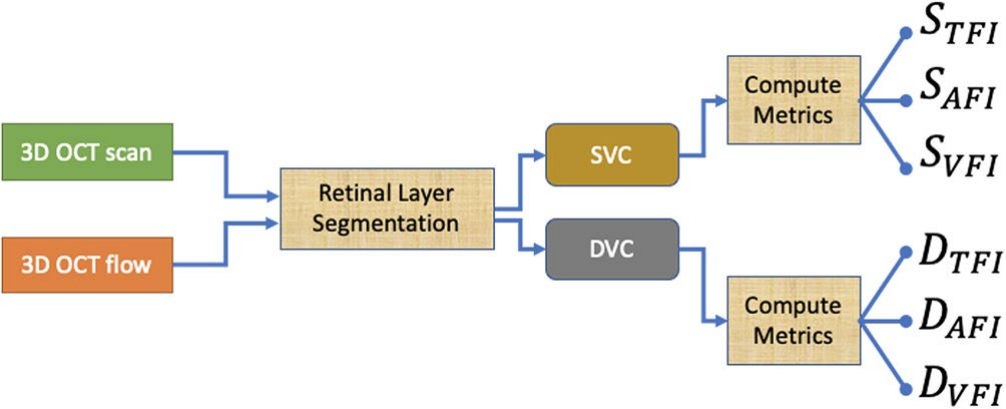
Workflow for computing TFI, AFI and VFI OCTA metrics. *OCT* optical coherence tomography, *OCTA* optical coherence tomography angiography, *SVC* superficial vascular complex, *DVC* deep vascular complex, *TFI* total flow intensity. *AFI* active flow intensity, *VFI* volume-related flow intensity.

The VFI metric is a measure of the ratio between a retinal layer TFI and the corresponding volume that does not exhibit flow activity (i.e. volume with corresponding voxel with null flow information). It is computed using the formula

$${I}_{VFI}=\frac{1}{{w}_{\mathrm{VFI}}\overline{F }}\sum_{i=1}^{\mathrm{V}}f{\left({x}_{i},{y}_{i},{z}_{i}\right)}^{2}$$

where $V$ is the number of voxels $P\left({x}_{i},{y}_{i},{z}_{i}\right)$ in the retinal layer under test, $\overline{F }$ is the number of voxels where $f\left(P\right)=0$ (i.e. the OCT blood flow does not exhibit any activity), $f({x}_{i},{y}_{i},{z}_{i})$ is the blood flow intensity of these voxels, ${w}_{F}$ is scaling parameter introduced for controlling the index range. The theoretical range for the VFI metric is ${I}_{VFI}\epsilon ]\mathrm{0,1}[$. When ${I}_{VFI}\to 0$, there is no flow activity or the flow activity is marginal with respect to the ‘free space’ ($\overline{F }$), while when ${I}_{VFI}\to 1$, the blood vessels flow activity is constrained by shortage of free space. The entire process is schematically shown in Fig. 6.

Our experiment was based on the comparison between VD, TFI, AFI and VFI metrics obtained from healthy volunteers and STGD patients.

All the statistical analyses were performed by using SPSS software package (SPSS, Chicago, Illinois, USA). Statistically significance was set to p < 0.05.

## Results

**Figure 7:**
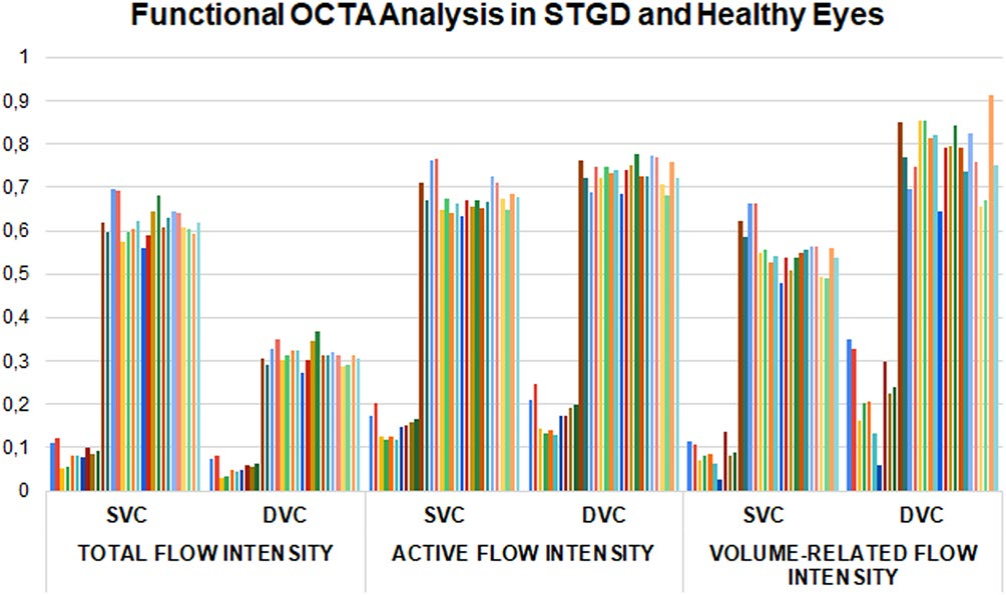
Functional OCTA analysis in STGD and healthy eyes.

We included 20 eyes of 20 healthy subjects (12 males) and 10 eyes of 10 STGD patients (6 males). Clinical and OCTA quantitative metrics are extensively reported in Table 1. Looking at TFI, AFI and VFI, all these functional OCTA parameters resulted significantly worse in STGD than controls (Fig. 7 and Table 1).

**Table 1:** Clinical Data and OCTA Quantitative Metrics in STGD and Healthy Subjects.

Parameter	Age	BCVA LogMAR	
STGD	36 ± 6	0.41 ± 0.12	
Healthy subjects	34 ± 5	0.0 ± 0.0	
p value	> 0.05	< 0.001	

Parameter	TFI SVC	TFI DVC	p value SVC vs DVC
STGD	0.09 ± 0.02	0.05 ± 0.02	< 0.001
Healthy subjects	0.62 ± 0.04	0.31 ± 0.02	< 0.001
p value	< 0.001	< 0.001	

Parameter	AFI SVC	AFI DVC	p value SVC vs DVC
STGD	0.15 ± 0.03	0.17 ± 0.04	0.1
Healthy subjects	0.68 ± 0.04	0.73 ± 0.03	< 0.001
p value	< 0.001	< 0.001	

Parameter	VFI SVC	VFI DVC	p value SVC vs DVC
STGD	0.09 ± 0.03	0.22 ± 0.09	< 0.001
Healthy subjects	0.55 ± 0.05	0.78 ± 0.07	< 0.001
p value	< 0.001	< 0.001	

Parameter	VD SVC	VD DVC	p value SVC vs DVC
STGD	0.41 ± 0.02	0.24 ± 0.05	< 0.001
Healthy subjects	0.41 ± 0.02	0.43 ± 0.01	< 0.001
p value	> 0.05	< 0.001	

*SVC* superficial vascular complex, *DVC* deep vascular complex, *TFI* total flow intensity, *AFI* active flow intensity, *VFI* volume-related flow intensity, *VD* vessel density.

**Figure 8:**
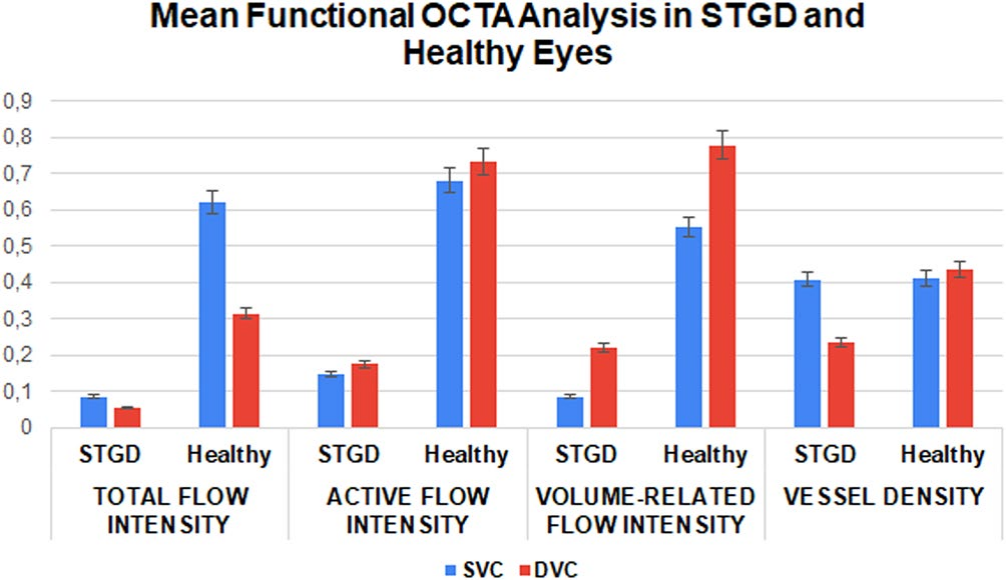
Mean functional OCTA analysis in STGD and healthy eyes.

In healthy subjects, TFI resulted significantly higher in SVC, compared to DVC (p < 0.01); on the contrary, DVC disclosed significantly higher AFI and VFI values than SVC (p < 0.01) (Fig. 8).

These statistically significant differences persisted also in STGD eyes, although AFI resulted comparable between SVC and DVC (p > 0.05) (Table 1). Moreover, the histogram plot showed different behavior of the functional OCTA metrics changes in STGD (Fig. 6). With respect to TFI, this resulted remarkably higher in SVC of healthy controls. This difference was maintained in STGD, although the loss of SVC component resulted more pronounced than DVC one. On the contrary, AFI and VFI loss were characterized by the same proportion between SVC and DVC contribution, with respect to controls.

It is worth notice that VD resulted higher in DVC than SVC in healthy eyes. This metric disclosed an almost exclusive involvement of the DVC in STGD eyes (Table 1 and Fig. 6). The correlation analysis revealed statistically significant correlations between TFI and AFI as well as between AFI and VFI. Moreover, VD correlated with TFI and AFI. No significant correlations were found between TFI and VFI. Significant values are disclosed in Table 2.

**Table 2:** Quantitative OCTA metrics correlation analysis.

TFI SVC	Parameter	TFI DVC	AFI SVC
	Tau Kendall coeff	0.589	0.442
	p value	< 0.001	< 0.001

TFI DVC	Parameter	AFI DVC	VD DVC
	Tau Kendall coeff	0.474	0.442
	p value	< 0.001	< 0.001

AFI SVC	Parameter	VFI SVC	
	Tau Kendall coeff	0.611	
	p value	< 0.001	

AFI DVC	Parameter	VFI DVC	VD DVC
	Tau Kendall coeff	0.495	0.421
	p value	< 0.001	0.009

*SVC* superficial vascular complex, *DVC* deep vascular complex, *TFI* total flow intensity, *AFI* active flow intensity, *VFI* volume-related flow intensity, *VD* vessel density.

## Discussion

In the present paper, we described three novel OCTA quantitative parameters, named TFI, AFI, and VFI. These metrics aimed to provide novel tools for gathering relevant retinal volumetric functional perfusion information, with respect to the morphological information provided by VD.

As previously described in the methods section, TFI represents the measure of the overall amount of volumetric blood flow interesting SVC and DVC, respectively. TFI resulted significantly higher in SVC than in DVC. This result is consistent with the specific morpho-functional features characterizing SVC. Indeed, the superficial retinal plexus is bigger than DVC, made by thicker capillaries making an intricate network of anastomoses among them, and creating connections with DVC capillaries^14,16^.

On the contrary, DVC involves a smaller volume of the retina and is made by thinner capillaries, with a less represented perfusion amount than SVC^14^. Due to these features, it is plausible to find an overall perfusion amount, represented by TFI, bigger in SVC than in DVC.

It is worth of notice that, both in previous papers and in the present study, healthy VD values resulted comparable or higher in DVC than in SVC^4,5,7,10,11,16^. This is a potential contradiction with what is discussed above about the morpho-functional features of SVC and DVC, but it can be explained by the fact that VD calculation does not take into consideration the different retinal volumes interested by the two plexa, thus causing a potential overestimation of the DVC. Indeed, the VD calculation is based on the planar enface reconstruction of the DVC capillaries, thus not considering the volumetric contribution.

From this point of view, TFI can provide likely information regarding the perfusion status of SVC and DVC. In STGD, TFI resulted significantly reduced both in SVC and DVC; on the contrary, VD resulted reduced only in DVC. The VD results agreed with previous investigations disclosing a significant and exclusive involvement of DVC, followed by SVC significant impairment occurring in later STGD stages^11,17^.

However, VD can be considered just a morphological parameter describing the anatomic loss of retinal capillaries. This interpretation justifies the later onset of SVC involvement since DVC is known to be earlier affected by blood flow impairment causing capillaries closing. However, it is assumable that STGD induces a global functional impairment both of inner and outer retina, thus leading to reduced perfusion amount both of SVC and DVC. Indeed, in advanced stages, all retinal layers resulted significantly impaired.

In the scenario comprising that retinal functional impairment precedes anatomic one, TFI could represent a useful functional parameter for the earlier detection of retinal blood flow reduction. Its significant impairment, detected also in SVC, may be explained by the fact that TFI metric adopts a volumetric approach and it directly assesses the changes of the blood flow signal. On the contrary, VD is less sensitive in detecting blood flow signal reductions.

AFI is a parameter that considers only the signal of voxels interested by blood flow perfusion. This parameter was developed to provide a pure measure of perfusion, without considering avascular volume. This latter volume was included in VFI calculation, which represents the ratio between TFI of a given retinal plexus and the corresponding volume that does not exhibit blood flow phenomenon. Since AFI represents pure perfusion whereas VFI was built to consider somehow the perfusion distribution within SVC or DVC retinal volumes, with respect to the not vascularized retinal regions, these two parameters have different functional meaning.

However, they disclosed a close relationship between them, since the starting common point is represented by the homogeneity of the blood flow signal interesting SVC and DVC. Since DVC is anatomically smaller than SVC, it may be less probable to find a high number of coexisting high intensity and low intensity voxels. This consideration leads to the production of a more homogeneous signal in DVC, traduced in higher AFI and VFI values than those disclosed by SVC.

Indeed, a wider anastomotic network, which characterizes SVC, might lead to a higher presence of capillaries disclosing different numbers of erythrocytes and different blood flow speeds^14^. Both conditions represent the basis of more heterogeneous intensity values of SVC voxels, compared to DVC ones, thus causing a lowering of the final calculated AFI and VFI values.

On the other side, we cannot exclude that the higher AFI and VFI values disclosed by DVC might be related to higher metabolic demand provided by the retinal layers perfused by this vascular network. From this point of view, further studies should be conducted to assess differences in terms of energetic demands among all retinal cytotypes.

The practical application of AFI and VFI metrics is disclosed in STGD eyes. Indeed, both parameters showed significant reductions, compared to healthy eyes, both in SVC and DVC. This first finding agreed with TFI metric. However, dissimilarly from TFI, both AFI and VFI preserved the starting difference between SVC and DVC values, maintaining higher values in DVC. This means that the overall reduction of active voxels is similar between SVC and DVC, as represented by AFI. Moreover, a reduction of the retinal vascular supply is associated by a similar reduction of the non-vascular compartment, as disclosed by VFI.

All these morpho-functional findings were not provided by VD, which was simply able to document a decrease of the DVC. On the other side, our new metrics raised interesting new insights about the assessment of the involvement both of vascular and non-vascular retinal structures. Because of the cross-sectional nature of our study, we were able to document only a simultaneous impairment both of vascular and non-vascular retinal compartments. Further prospective studies, using similar quantitative approaches, might be able to establish the timing and the amount of involvement of both compartments, thus providing new diagnostic approaches to monitor STGD and other retinal diseases.

Although our study should be intended as a first assessment regarding the usage of the new proposed OCTA quantitative metrics, the present findings already suggested a clinical relevance of TFI, AFI and VFI. TFI was built to perform the overall quantitative assessment of the amount of perfusion of SVC and DVC. From this point of view, it can be considered the global estimate of the amount of blood flow interesting the whole segmented retinal region (SVC or DVC). AFI was built to perform the exclusive quantitative analysis of only the voxels resulting interested by OCTA signal. From this point of view, AFI can be interpreted as the absolute estimates of SVC and DVC perfusion. VFI introduces the volume as a variable, in order to perform a quantitative estimate of intraretinal blood flow pondered for the perfused volume. In terms of application in clinical practice, TFI can be used to assess if the overall retinal perfusion amount is within the range of normality or not. AFI can be used to selectively quantify the vascular impairment occurring in a given pathological condition. VFI can be sensitive in distinguishing if the retinal impairment is mainly focused on the vascular or in the non-vascular compartments.

We are aware that our investigation might be potentially affected by several limitations. First of all, due to the feasibility nature of our study, we included a relatively low number of healthy and STGD eyes. In particular, we performed just an overall investigation of TFI, AFI and VFI alterations in STGD, merely intended as an age-matched group of eyes affected by a retinal disease to be compared with healthy volunteers, without considering STGD stages as in previous investigations^18^. From this point of view, future studies should be focused on the application of TFI, AFI and VFI metrics in different stages of STGD. Furthermore, we are aware that grades of blood flow above or below the OCTA machine threshold will be represented by similar intensity voxels. Although this potentially makes our metrics prone to under/overestimations, the comparison of the values between healthy and pathological conditions should not be remarkably affected. From this point of view, our metrics will benefit from advances in OCTA technology, making possible to obtain even more detailed information about the blood flow features in healthy and pathological retinal conditions. In terms of artifacts affecting OCTA analyses, our new metrics may be still artifacts prone. However, if compared to VD, the proposed metrics (TFI, AFI and VFI) can be considered remarkably less affected by potential biases related to the proper enface reconstruction of retinal vascular plexa, including for example blinking, motion and segmentation artifacts. Moreover, our metrics did not suffer from possible limitations related to the binarization process, which strictly depends on the threshold applied to SVC and DVC reconstructions. On the other side, accurate volume pre-processing has a strong impact on the reproducibility and repeatability of the proposed metrics. Low quality volume registration may provide unreliable results, and therefore a visual inspection of the registered volume is required before computing the proposed metrics. Since ours was a first investigation, we included only one high-quality OCTA volume for each eye. For this reason, we cannot provide accurate reproducibility and repeatability data. Since the proposed quantitative approach is mainly based on automatic steps, with the only exception of volume registration check, we can consider our methodology less operator dependent than other OCTA metrics, which require careful layers’ segmentation and binarization threshold choosing. However, further larger validations and tests are required to reach more definite conclusions about the applicability of TFI, AFI and VFI in clinical and research settings.

For all these reasons, TFI, AFI and VFI might represent more reliable quantitative biomarkers for the study of retinal vascular features. However, further studies are warranted to validate our metrics and to reinforce their interpretation both in healthy and pathologic conditions, with the help of also histologic approaches. Furthermore, TFI, AFI and VFI metrics values may be influenced both by the number of motion particles passing within the retina and the blood flow speed. Further studies are warranted to assess the relationship between TFI, AFI and VFI metrics changes, and the specific cause of these modifications.

Lastly, because of the intrinsic difficulty to properly detect the choriocapillaris layer, because of the extremely low thickness, we decided to exclude choriocapillaris from the present study. Further investigations are warranted to calculate TFI, AFI and VFI also in the choriocapillaris.

In conclusion, our study proposed three new OCTA quantitative parameters, namely TFI, AFI and VFI, which were built to provide direct functional information regarding the blood flow features of SVC and DVC. These metrics were developed in an attempt to study OCTA data from new points of view, thus representing potentially useful functional biomarkers to be used together with the already adopted morphological parameters, such as VD.
